# Online Adaptive Radiation Therapy: Implementation of a New Process of Care

**DOI:** 10.7759/cureus.1618

**Published:** 2017-08-27

**Authors:** James Lamb, Minsong Cao, Amar Kishan, Nzhde Agazaryan, David H Thomas, Narek Shaverdian, Yingli Yang, Suzette Ray, Daniel A Low, Ann Raldow, Michael L. Steinberg, Percy Lee

**Affiliations:** 1 Department of Radiation Oncology, University of California, Los Angeles; 2 Department of Radiation Oncology, University of Colorado, Denver

**Keywords:** mri, on-line adaptive radiotherapy

## Abstract

Onboard magnetic resonance imaging (MRI) guided radiotherapy is now clinically available in nine centers in the world. This technology has facilitated the clinical implementation of online adaptive radiotherapy (OART), or the ability to alter the daily treatment plan based on tumor and anatomical changes in real-time while the patient is on the treatment table. However, due to the time sensitive nature of OART, implementation in a large and busy clinic has many potential obstacles as well as patient-related safety considerations. In this work, we have described the implementation of this new process of care in the Department of Radiation Oncology at the University of California, Los Angeles (UCLA). We describe the rationale, the initial challenges such as treatment time considerations, technical issues during the process of re-contouring, re-optimization, quality assurance, as well as our current solutions to overcome these challenges. In addition, we describe the implementation of a coverage system with a physician of the day as well as online planners (physicists or dosimetrists) to oversee each OART treatment with patient-specific ‘hand-off’ directives from the patient’s treating physician. The purpose of this effort is to streamline the process without compromising treatment quality and patient safety. As more MRI-guided radiotherapy programs come online, we hope that our experience can facilitate successful adoption of OART in a way that maximally benefits the patient.

## Introduction

Onboard magnetic resonance imaging (MRI) guidance allows for the accurate visualization of day-to-day changes in size, position and deformation of tumors and organs at risk, thereby enabling online adaptive radiotherapy (OART). As of 2017, MRI-guided OART cases are being treated at nine cancer centers worldwide using the ViewRay MRIdian® system. Initial experience has indicated promising utility in the abdominopelvic region where this strategy can minimize irradiation of the bowel and stomach based on daily anatomic variations [1-2]. New and innovative processes, techniques, and workflows are required to maximize the safety, efficiency and effective use of this new radiation therapy paradigm. Beginning in July 2015, a program was implemented to establish MRI-guided OART at our institution. Since then, we have treated over 80 online adaptive stereotactic body radiotherapy (SBRT) fractions. In this white paper, we share our experience on the implementation of this new process of care in radiation therapy to guide future centers. Key quality considerations include:

- Streamlining the process and reducing the time consumed

- Obtaining the best possible re-planned dosimetry given the changes in patient anatomy

- Minimizing the probability of human errors during the time-condensed planning process through error avoidance and error detection techniques

## Technical report

Adaptive workflow overview

Our online adaptive radiotherapy workflow is a choreographed process involving contributions from the radiation therapist, the medical physicist, and the physician. The process is described briefly as follows. First, the radiation therapists bring the patient into the room, perform initial setup, acquire a volumetric MRI suitable for both target alignment and treatment planning, align the treatment target in the image-guided radiotherapy workspace, and then page the covering physician and adaptive planner. The adaptive planner may be a physicist or dosimetrist. Deformable-registration based auto-contouring is initiated, followed by manual edits of auto-generated critical structure contours. Critical structure contours are typically edited by the adaptive planner and always reviewed by the attending physician. The gross tumor volume is propagated rigidly (not deformed) and may be edited by the covering physician. Derived structures are generated, such as planning target volumes and tuning structures. The dose is recalculated using an electron density image derived by registration of the initial plan electron density to the setup image. The dose which would be delivered if the plan is not adapted, referred to as the “predicted dose”, is evaluated using dose-volume histograms based on the re-contoured structures. Based on the predicted dose and the anatomy visualized in the setup image, a decision is made whether to treat as-is or to adapt. Attending physicians specify quantitative adaption criteria per plan, in most cases requiring adaptation if the volume of small bowel or stomach receiving 35 Gy exceeds 0.5 cubic centimeters. If the decision is made to adapt, the treatment planning system performs intensity-modulated radiotherapy (IMRT) optimization with the same structure weights and beam angles as the offline plan (only the structures themselves, as well as the electron density map, having changed). Beam angles and structure weights can be edited if needed, but usually, are not edited because of the corresponding increase in time required. Dosimetry of the adaptive plan is evaluated and a decision is made whether to treat the adaptive plan, treat the initial plan, or abort the fraction. Finally, gating parameters are set, if applicable, and the treatment is initiated. The gating workflow has been described elsewhere [3]. Table 1 shows a list of actions as well as the staff who perform the action and who review the results of the action.

**Table 1:** Workflow actions. Adaptive workflow actions and corresponding staff roles.

Action	Performed by	Reviewed by
Acquire setup imaging and align patient	Therapist	Physician, physicist
Critical structure re-contouring	Physicist/dosimetrist	Physician
Gross tumor volume contour, as needed	Physician	Physicist/dosimetrist
Create derived contour structures	Physicist/dosimetrist	Physician
Pre-adaptation evaluation	Physician	Physicist/dosimetrist
Plan re-optimization	Physicist/dosimetrist	Physician
Critical structure dose evaluation	Physician	Physicist/dosimetrist
Quality assurance checks	Physicist	Physician
Configuration of gating and beam-on	Therapist	Physician, physicist

Physician and planner coverage and communication scheme

In an initial ramp-up period, a core group of one physician and three physicists performed all adaptive planning and quality assurance (QA) tasks. As the number of adaptive cases began to increase, a rotating coverage model was implemented. Adaptive physician coverage was delegated to the department’s doctor of the day (DOD). The DOD was an existing role in the department prior to implementation of MRI-guided radiotherapy, and is responsible for coverage of SBRT cases on all treatment machines. Adaptive planner coverage was delegated to a dedicated rotation of physicists and dosimetrists. If the adaptive planner was a dosimetrist, the presence of the departmental “physicist of the day” in the treatment control area was also required. An in-service lecture was provided to train all DODs in the adaptive workflow and contouring and plan evaluation tools. Adaptive planners were chosen from the pool of off-line ViewRay planners and were trained using the ViewRay treatment simulator. All departmental qualified medical physicists were trained in the use of the online quality assurance tool.

**Figure 1:**
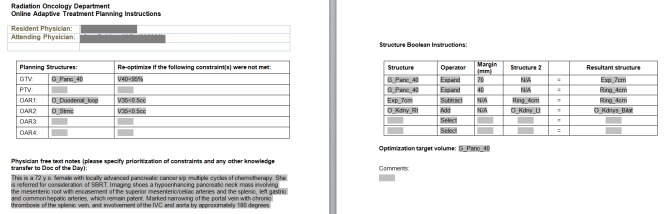
Communication forms. Templated communication forms for covering physician (A) and physicist (B).

In order to facilitate knowledge transfer between the attending physician and the covering physician, a document template was created in the ARIA electronic medical record system. The attending physician enters a list of dose constraints. If the listed dose constraints are not achieved, plan adaptation is triggered, minimizing the judgement calls for the DOD who may not be familiar with the clinical history and has limited time to evaluate adaptive planning. The treatment planning portion of the document gives instructions for re-creating any derived structures used in plan optimization (PTV, ring structures, etc.), as well as the target volume used for optimization and a free text notes field (Figure 1).

Treatment time considerations

Timing measurements were performed during 22 adapted abdominal and pelvic SBRT fractions. Table 2 shows the time taken in various steps of the process between the patient entering the vault and the commencement of treatment delivery. The median time to beam commencement was 54 minutes (range 34-99 minutes). Typical total treatment time for adaptive gated SBRT treatment including beam delivery time is approximately 90 minutes. Adaptive SBRT cases are scheduled in 120 minutes time slots to account for the substantial variance in time.

**Table 2:** Measured time. Measured time for each step in the adaptive process.

Process step	Median (min-max) time (minutes)
Room patient and acquire image used for re-plan	9 (5-30)
MD approval of initial setup	7 (3-18)
Adaptive re-contouring	10 (5-22)
Adjust plan and perform quality assurance (QA)	14 (8-40)
Total	54 (34-99)

The following were identified as causes of extended time:

1. Planner or physician not responding to pages in a timely manner

2. Planner or physician unfamiliar with online planning tools

3. Contouring more anatomy than necessary or at a higher level of detail than necessary

4. Contouring error made and not noticed until time of adaptive plan evaluation

5. Needing to make an in-depth plan modification (changing structure optimization weights)

Adaptive re-contouring

**Figure 2:**
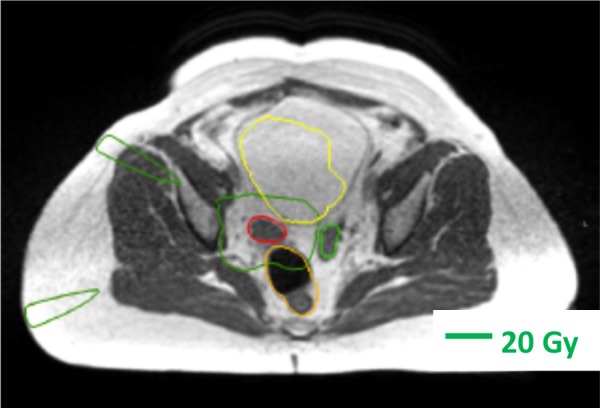
Adaptive re-contouring. In this case, deformably propagated contours on the bladder wall distal to the target were not corrected because the un-contoured portion was expected and confirmed to receive less than 20 Gy. The dark green line indicates the 20 Gy isodose contour. The red line indicates the planning target volume contour. The light green line indicates the small bowel contour. The orange line indicates the rectum contour. The yellow line indicates the bladder contour.

The deformable contour propagation provided by the ViewRay treatment planning system (TPS) facilitates adaptive re-contouring. However, considerable manual editing is still needed. It is often preferable to delete the stomach contour and re-contour it from scratch due to the apparent inability of the auto-contouring algorithm to adapt to large changes in organ size. Because the covering physician may not be the patient’s attending physician and may not be a specialist in the patient’s type of cancer, we do not allow the TPS to deform the gross tumor volume (GTV). We require the GTV to be aligned rigidly and edited as necessary by the physician. All structures weighted in the optimization and/or used for evaluation are contoured in slices containing the target plus a 1 cm superior/inferior margin. To save time, boundaries are not precisely adjusted in regions where the corresponding dose volume histogram (DVH) points would not be actionable (Figure 2). It is recommended that the planner and physician review each other’s work in real-time in order to catch mistakes in this time condensed treatment planning process. In our current workflow, the distance from the target beyond which contouring precision is lowered is left to the intuition of the planner. After adaptive dose is calculated, it is verified by the planner and the physician that structures are contoured with sufficient accuracy to inform all actionable dose constraints.

Strategies to minimize re-planning time

**Figure 3:**
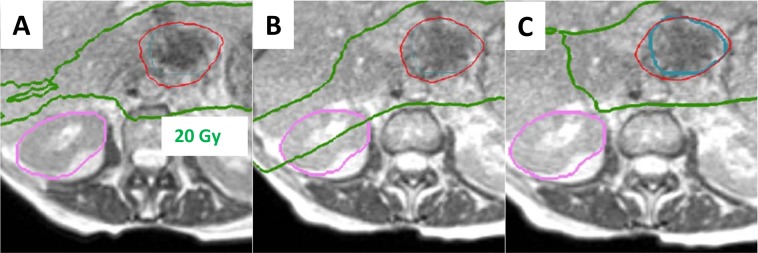
Challenging pancreatic stereotactic body radiotherapy (SBRT). A challenging pancreatic SBRT case treated adaptively. The 40 Gy prescription isodose line is shown in red, and the 20 Gy isodose line is shown in green. In the initial plan (A), the kidney was not weighted in the optimization, but received less than tolerance dose. During the first adaptive fraction (B), a change in the bowel near the target caused the optimizer to turn a beam on through the kidney. Subsequently, an optimization weight was added to the kidney, which improved the dosimetry (C) at the expense of increased planning time.

At our institution, MRIdian SBRT cases are typically planned using 15-18 equally-spaced co-planar beams. In order to make adaptive re-planning faster and avoid the need to change beam angles and/or structure weights, the non-specific normal tissue constraint is heavily weighted in the optimization. Derived optimization structures such as rings or tuning target volumes are avoided if possible. Adaptive planning benefits from adherence to standardized structure naming conventions since the covering physician and planner may not be familiar with the strategies and goals of the initial approved plan. Extraneous structures not used in optimization or evaluation are removed from the plan. Particular care is taken to remove unused target structures and/or abandoned attempts at different expansion margins. Finally, all structures co-planar with the target should have some weighting applied in the optimization; otherwise, an organ at risk not accounted for may receive unnecessarily higher dose when the plan is adaptively re-optimized (Figure 3).

Patient-specific IMRT QA

The MRIdian and its online adaptive treatment process are intended to be used with IMRT. Pre-treatment phantom QA is not possible for online adaptive due to the presumption that moving the patient from the treatment table invalidates the adaptive plan by potentially altering the patient’s anatomic configuration. IMRT QA is performed by comparing the TPS dose to a plan recalculation using a secondary Monte Carlo tool provided by ViewRay. The secondary Monte Carlo tool incorporates a beam model equivalent to the one in the TPS and recalculates the planned dose using a file exported by the TPS containing MLC leaf positions and beam on times. The fact that the secondary Monte Carlo uses an equivalent machine model as the TPS, and was written by the same team of scientists and engineers, represents a limitation, because any mistakes in the TPS are likely to be replicated in the secondary Monte Carlo. In order to partially mitigate this limitation, phantom-based IMRT QA of a standard, highly modulated plan is performed on a weekly basis to assess the stability of machine performance. During our initial experience with adaptive therapy, the first 20 adapted fractions were retrospectively delivered to an IMRT QA phantom and passed with typical rates (i.e., >95% gamma pass rate with 3%/3 mm criteria).

Our initial experience with online adaptive was facilitated by a software “wrapper” provided by Washington University in Saint Louis (currently available from ViewRay), which was used to drive the secondary Monte Carlo tool and analyze and present the results [1]. Subsequently, we developed our own analysis wrapper (as described below), incorporating code written at the University of Wisconsin under the GNU General Public License. The electron density used for adaptive dose calculation by the treatment planning system is derived from the initial plan’s electron density via image registration of the respective MRI images. The adaptive plan’s electron density must be visually reviewed prior to treatment.

Plan consistency checks

**Figure 4:**
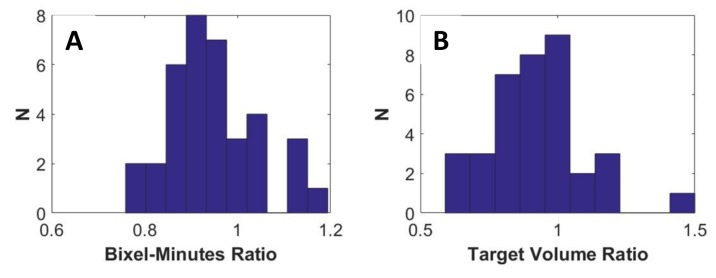
Bixel minute ratio. Bixel-minute ratio (A) and target contour volume ratio (B) in 40 adapted fractions. Variations in target structure volume resulted from increased and decreased to target volume margins based on physician judgement and proximity to dose limiting structures.

Off-line IMRT planning incorporates a number of implicit and explicit consistency checks and review of plans by physicists uninvolved in the planning. In online planning, some consistency checks must be automated. For example, in our clinic, uninvolved physicists review the correctness of contours and review that the plan dosimetry is consistent with similar plans based on experience. Such checks are too time-consuming in an online adaptive workflow. Software was developed in order to evaluate the consistency of contour volumes, segment times, and DVH points. The ‘bixel-minutes’, or sum of beam on time multiplied by segment area (a measure of integral dose) is tracked as a safety check. Figure 4 shows histograms of the ratio of adapted to initial bixel-minutes and target structure volume, respectively. If the bixel-minutes ratio is outside the range 0.9-1.1, further investigation is performed by the physicist. For example, it is verified that the ratio agrees with an intended change in the target volume or dose normalization of the adapted plan. If a target volume ratio is outside the range 0.9-1.1, the physicist confirms that the physician intended that degree of change. Additionally, these ratios are examined because they may be sensitive to unexpected error modes.

## Discussion

Our adaptive radiotherapy workflow is intended to minimize resources required while maintaining the highest level of treatment quality, thereby enabling us to bring the benefits of adaptive radiotherapy to the largest number of patients possible. Development of the optimal workflow remains a work in progress and is a collaborative effort between several early-adopting clinics in both the low-field and high-field MRI-guided radiotherapy communities. As the online adaptive radiotherapy process continues to be refined, we expect that the development of the following improvements will be important:

1. A fast and user-friendly interface to choose the base plan from all previously treated plans for a given patient in a given course.

2. Improvement in image quality that would aid in the accuracy and speed of auto-segmentation software.

3. An adaptive optimizer that runs multiple plans in parallel with a range of critical structure weights, and lets the user choose which one he/she likes best.

4. A decision support tool for physicians to evaluate a fully adaptive course.

## Conclusions

MRI-guided online adaptation is a new paradigm that holds promise to improve the therapeutic ratio of abdominopelvic and other sites of radiotherapy because daily variations in targets and critical normal structures can be visualized and compensated. In this whitepaper, we have described our clinic’s implementation of an adaptive workflow in the hopes that it may be useful to other clinics adopting MRI-guided radiotherapy.
